# miR-378f targets CAB39 to regulate autophagy in chemotherapy-induced peripheral neuropathy

**DOI:** 10.3389/fnmol.2026.1958322

**Published:** 2026-10-07

**Authors:** Yeeun Kim, Myeongguk Jeong, Hyeokjin Kwon, Jungho Kim, Yeongdon Ju, Do-Young Kang, Aelee Jang, Go-Eun Choi

**Affiliations:** 1 Department of Translational Biomedical Sciences, Graduate School of Dong-A University, Busan, Republic of Korea; 2 Department of Clinical Laboratory Science, College of Health Sciences, Catholic University of Pusan, Busan, Republic of Korea; 3 Research Center for Endocrine and Metabolic Diseases, Convergence Medical Institute, Chungnam National University, Daejeon, Republic of Korea; 4 Next-Generation Industrial Field-Based Specialist Program for Molecular Diagnostics, Brain Busan 21 Plus Project, Graduate School, Catholic University of Pusan, Busan, Republic of Korea; 5 Department of Biomedical Laboratory Science, Gimcheon University, Gimcheon, Republic of Korea; 6 Department of Nuclear Medicine, Dong-A University College of Medicine, Busan, Republic of Korea; 7 Department of Nursing, University of Ulsan, Ulsan, Republic of Korea

**Keywords:** autophagy, calcium-binding protein 39, chemotherapy-induced peripheral neuropathy, microRNA, neuroregeneration

## Abstract

Chemotherapy is the most common cancer therapy due to its ability to effectively destroy cancer cells. However, peripheral neuropathy is a notable side effect, which has no clearly established prevention or treatment methods. Although various therapeutic strategies using microRNAs (miRNAs) have been developed for a wide range of diseases, the role of miRNAs in neuroregeneration has yet to be elucidated. Our group previously identified five differentially expressed miRNAs in the plasma of patients with gastric cancer receiving XELOX (capecitabine and oxaliplatin) chemotherapy. Among these, miR-378f alone showed a significant correlation with peripheral neuropathy severity, providing the rationale for the present study. Bioinformatic analysis further predicted calcium-binding protein 39 (CAB39), a regulator of autophagy, as a shared target of these miRNAs. The downregulation of miR-378f increased CAB39 expression, activated AMPK and inhibited mTOR. Furthermore, changes in autophagy-related markers were observed, including increased Beclin-1 expression, elevated LC3-I to LC3-II conversion, and decreased p62 expression. The findings of the present study suggest that autophagy regulation by miR-378f may be a novel diagnostic and therapeutic target for peripheral neuropathy.

## 1 Introduction

Chemotherapy-induced peripheral neuropathy (CIPN) is a frequent and dose-limiting adverse effect of several anticancer agents and can persist long after treatment, substantially impairing patients’ quality of life (Lee et al., 2024; Amarelo et al., 2025; Tao et al., 2025). CIPN may also necessitate chemotherapy dose reduction or discontinuation, potentially compromising treatment efficacy (Chen et al., 2024). XELOX, consisting of capecitabine and oxaliplatin, is widely used for advanced gastric cancer, but oxaliplatin is well recognized for its neurotoxicity (Hwang et al., 2017; Zhou et al., 2026). Therefore, understanding the molecular responses associated with oxaliplatin-induced peripheral nerve injury is important for identifying mechanisms that may contribute to neuronal protection and repair.

MicroRNAs (miRNAs) are small non-coding RNAs, typically composed of 21s-23 nucleotides, that regulate gene expression by binding to complementary sequences within target mRNAs, thereby suppressing translation or promoting mRNA degradation (Cai et al., 2009; Dexheimer and Cochella, 2020). Dysregulation of specific miRNAs has been implicated in diverse pathological processes, including cancer, neurological disorders, and inflammation. For example, circulating miR-221 and miR-222 have been investigated as biomarkers in breast cancer, whereas miR-124, miR-146a, and miR-155 have been associated with neuronal differentiation, neuroinflammation, and inflammatory signaling pathways (Kim et al., 2019; Kim and Cho, 2021; Das and Rao, 2022; Smolarz et al., 2022; Li et al., 2023). More importantly in the context of the present study, accumulating evidence indicates that miRNAs can actively regulate neuronal stress responses and regenerative processes through modulation of oxidative stress, neuroinflammation, axonal growth, and autophagy-related pathways (Slota and Booth, 2019; Mohseni et al., 2024). miR-21 has been shown to promote Schwann cell proliferation and axonal regeneration following peripheral nerve injury, whereas miR-1265 regulates autophagy by targeting CAB39 and modulating AMPK/mTOR signaling (Xu et al., 2019; Ning et al., 2020). Representative miRNAs implicated in neuronal regulation, inflammation, autophagy, and peripheral nerve repair are summarized in Supplementary Table S1. Thus, circulating miRNA expression patterns may reflect disease-associated molecular changes, while individual miRNAs can actively regulate neuronal stress and repair processes (Borger et al., 2022; Rodwin et al., 2022).

Although oxaliplatin-induced peripheral neuropathy is not specific to gastric cancer, studying a relatively homogeneous cohort of patients with advanced gastric cancer receiving the same oxaliplatin-based regimen may reduce heterogeneity in cancer type, treatment schedule and drug exposure, thereby facilitating the interpretation of CIPN-associated miRNA alterations. Our previous study employing small RNA sequencing of plasma samples from patients with gastric cancer identified five differentially expressed miRNAs associated with chemotherapy exposure: hsa-miR-4666a-3p, hsa-miR-885-5p, hsa-miR-200c-3p, hsa-miR-378f and hsa-miR-27b-3p (Ju et al., 2022). Critically, subsequent RT-qPCR validation demonstrated that miR-378f was the only candidate among the five to show a statistically significant correlation with peripheral neuropathy severity, establishing it as the most biologically relevant target for mechanistic follow-up. Notably, calcium-binding protein 39 (CAB39), a gene involved in the regulation of cellular proliferation and autophagy, was predicted by TargetScan analysis to be a potential target of miR-378f (Li et al., 2018; Xu et al., 2019). These findings prompted further investigation into whether miR-378f regulates autophagy-related signaling in nerve cells through CAB39.

Macroautophagy, hereafter simply termed autophagy, is a genetically controlled and dynamic cellular process involving the creation of autophagosomes, which are double-membraned vesicles that sequester cytoplasmic materials (Cao et al., 2021; Liu et al., 2023; Yamamoto et al., 2023). Autophagy is primarily considered a protective cellular mechanism that promotes survival under stress by supplying nutrients and removing damaged organelles (Jing and Lim, 2012; Yon et al., 2023). In the peripheral nervous system, autophagy plays a particularly important role in nerve repair, as peripheral nerves retain a notable capacity for regeneration following injury (Scheib and Höke, 2013). The activation of autophagy in injured peripheral nerves has been shown to facilitate motor function recovery and myelin clearance, thereby supporting axonal repair, while also contributing to cellular survival and regenerative responses through the removal of damaged cellular components and interactions with neurotrophic and inflammation-related signaling pathways (Swiech et al., 2008; Levine et al., 2011; Huang et al., 2016; Liao et al., 2022). Of particular relevance, the activation of autophagy via mTOR inhibition has been shown to provide neuroprotection in the early stages of nerve injury and to support functional recovery (Swiech et al., 2008; Huang et al., 2016). The AMPK/mTOR pathway serves as a central regulatory axis of autophagy, with AMPK activation suppressing mTOR and consequently inducing autophagic flux (Wang et al., 2020; Wang et al., 2022). Furthermore, CAB39 is an established activator of AMPK (Tian et al., 2012; Zhuang et al., 2020), positioning it as a potential molecular link between miR-378f and autophagy-dependent neuroregenerative responses. The present study aimed to verify this hypothesis by investigating the functional relationship between miR-378f and CAB39 in neuronal cells, characterizing the downstream autophagy pathway, and validating the clinical relevance of this mechanism in patient-derived samples.

## 2 Materials and methods

### 2.1 Cell culture

SH-SY5Y cells (American Type Culture Collection; cat. no. CRL-2266) were maintained at 37˚C in a humidified incubator with 5% ${\mathrm{CO}}_{2}$ using a 1:1 mixture of Dulbecco’s modified Eagle’s medium and nutrient mixture F-12 (Gibco; Thermo Fisher Scientific, Inc.) supplemented with 10% fetal bovine serum, 100 U/mL penicillin and 100 µg/mL streptomycin.

### 2.2 miRNA lipofectamine transfection

The synthetic miR-378f mimic (cat. no. 4464066, Assay ID MC21788) and inhibitor (cat. no. 4464084, Assay ID MH21788) were purchased from Invitrogen (Thermo Fisher Scientific, Inc.). Corresponding negative controls, mimic-NC (Invitrogen; Thermo Fisher Scientific, Inc.; cat. no. 4464058) and inhibitor-NC (Invitrogen; Thermo Fisher Scientific, Inc.; cat. no. 4464076), served as the control RNAs in the experiments. SH-SY5Y cells were seeded at 1 × 10^6^/well in six-well plates and transfected with the miR-378f mimic, inhibitor, mimic-NC and inhibitor-NC using Lipofectamine RNAiMAX Transfection Reagent (Invitrogen; Thermo Fisher Scientific, Inc.; cat. no. 13778075). The lipofectamine transfection time evaluation was performed with mimic-NC and miR-378f mimic at 30 μM according to the manufacturer’s instructions. Once the optimal time was determined, the optimal concentration was further evaluated at 10, 30 and 60 μM. miR-378f expression levels were quantified using RT-qPCR.

### 2.3 Neurite length analysis

SH-SY5Y cells were observed under a cell culture microscope (CKX53; Olympus Corporation). Neurite length was measured by ImageJ software (v.1.52; National Institutes of Health), and each group is represented as the mean length of five neurites.

### 2.4 Total RNA isolation from cells

Total RNA extraction was initiated by aspirating the culture medium, followed by two washes with Dulbecco’s phosphate-buffered saline to remove residual media. After which, 1 mL TRIzol reagent was added and incubated for 1 h. Once the cells were completely lysed, 200 µL chloroform was added and the mixture was vortexed for 10 s. The lysates were left at room temperature (22˚C) for 5 min before being centrifuged at 12,000 × g for 10 min at 4˚C. A new microcentrifuge tube received the carefully separated upper aqueous phase and an identical volume of isopropanol was combined. The solution was subsequently held at 22˚C for 10 min, prior to being centrifuged at 4˚C for 10 min at a force of 12,000 × g. After discarding the supernatant, 1 mL 75% ethanol was used to wash the RNA pellet, which was subsequently centrifuged for another 5 min at 12,000 × g at 4˚C. Once the pellet was completely dry, it was dissolved in diethyl pyrocarbonate-treated water. The concentration and purity of the extracted RNA were determined using a NanoDrop 2000 spectrophotometer (Thermo Fisher Scientific, Inc.).

### 2.5 cDNA synthesis

cDNA was synthesized using the TaqMan MicroRNA Reverse Transcription Kit and TaqMan MicroRNA Assay (Applied Biosystems; Thermo Fisher Scientific, Inc.). Briefly, 3 μL 5X RT primers were mixed with 5 μL 2 ng/μL double-stranded template. The mixture was incubated at 85˚C for 5 min and 60˚C for 5 min, and quickly cooled on ice. After which, 7 µl Master Mix containing 100 mM deoxynucleoside triphosphates (with thymidine triphosphate), MultiScribe Reverse Transcriptase (50 U/μL), 10× Reverse Transcription buffer and RNase inhibitor (20 U/μL) was added to each reaction tube. Reverse transcription was initiated at 16˚C for 30 min, followed by a 30 min incubation at 42˚C and completed by heating at 85˚C for 5 min to complete cDNA synthesis.

### 2.6 RT-qPCR analysis

RT-qPCR was performed using a QuantStudio 7 Flex Real-Time PCR system (Applied Biosystems; Thermo Fisher Scientific, Inc.). TaqMan MicroRNA Assays (cat. no. 4427975) specific for hsa-miR-378f (Assay ID: 462794_mat) and U6 snRNA (Assay ID: 001973) were purchased from Thermo Fisher Scientific and used according to the manufacturer's instructions. In a total reaction volume of 20 μL, 1 μL TaqMan Small RNA Assay (20×), TaqMan Universal Master Mix II with UNG (10 μL), nuclease-free water (7.67 μL) and cDNA template (1.33 μL) were used. Each procedure included a negative control. The RT-qPCR reaction conditions were as follows: Initial step of 2 min at 50˚C and 10 min at 95˚C, followed by 40 cycles of 15 s at 95˚C and 1 min at 60˚C. Gene expression was analyzed using cycle threshold (Ct) values, and the relative expression level of miR-378f was quantified using the comparative ΔΔCt method (2^−∆∆Ct^) with U6 used as an endogenous control (Livak and Schmittgen, 2001).

### 2.7 Protein isolation and western blot analysis

Following transfection, cells were washed twice with phosphate-buffered saline and subsequently lysed in RIPA lysis buffer supplemented with protease and phosphatase inhibitors (GenDEPOT, LLC; cat. no. P3300). For 20 min at 4˚C, cell lysates were subjected to centrifugation at 12,000 × g. After which, the protein levels in the resulting supernatant were quantified with a BCA protein assay kit (Intron Biotechnology, Inc.). Protein (15 μg) was loaded for 10% sodium dodecyl sulfate-polyacrylamide gel electrophoresis and transferred to a polyvinylidene fluoride (PVDF) membrane. After which, the membranes were blocked with Smart Block 5 min Fast Blocking Buffer (Biomax) and incubated with the following primary antibodies overnight at 4˚C: Anti-Beclin1 (1:1,000; cat. no. 3495), anti-SQSTM1/p62 (1:1,000; cat. no. 39749), anti-MO25α/CAB39 (1:1,000; cat. no. 2716), anti-AMPKα (1:1,000; cat. no. 5832), anti-phospho-AMPKα (1:1,000; Thr172; cat. no. 2535), anti-mTOR (1:1,000; cat. no. 2983), anti-phospho-mTOR (Ser2448; 1:1,000; cat. no. 5536), anti-LC3A/B (1:1,000; cat. no. 12741) and anti-glyceraldehyde-3-phosphate dehydrogenase (GAPDH) (1:10,000; cat. no. 2118; all from Cell Signaling Technology, Inc.). Following primary antibody incubation, the PVDF membrane was treated with a horseradish peroxidase (HRP)-conjugated goat anti-rabbit secondary antibody (cat. no. 7074; Cell Signaling Technology, Inc.) at the same dilution as the corresponding primary antibody (1:1,000 or 1:10,000) for 1 h. Finally, proteins were detected by a ChemiDoc XRS + system (Bio-Rad Laboratories, Inc.) using an enhanced chemiluminescence kit. Protein levels were expressed relative to GAPDH levels.

### 2.8 Luciferase assay

To investigate the binding interaction with the 3’-UTR of CAB39, a potential target gene, plasmid constructs were generated using the pmirGLO Dual-Luciferase miRNA Target Expression Vector (Promega Corporation) based on predictions from TargetScan 7.2. After which, the SH-SY5Y cells were co-transfected with the reporter vector [CAB39 wild type (WT) or CAB39 mutant type (MUT)], miR-378f mimic, miR-378f inhibitor or negative control using Lipofectamine 2000 Transfection Reagent. A total of 24 h following transfection, both firefly and Renilla luciferase activities were evaluated utilizing the Dual-Luciferase Reporter Assay System (Promega Corporation).

### 2.9 Bioinformatics analysis

miRNA-target interactions were predicted using miRNet, which integrates experimentally validated target data from TarBase and miRTarBase. The predicted targets were visualized as an interaction network. Protein-protein interaction network analysis was subsequently performed using the Search Tool for the Retrieval of Interacting Genes/Proteins (STRING; version 12.0), with a minimum interaction confidence score of 0.4 applied as the threshold.

### 2.10 Clinical samples and CIPN grading

Between January and October 2021, blood samples were obtained from ten patients with advanced gastric cancer who received XELOX therapy (oxaliplatin and capecitabine) at Pusan National University Hospital (PNUH, Busan, Republic of Korea). Blood samples obtained from ten patients with gastric cancer who had received at least two cycles of XELOX chemotherapy were divided into two groups. Baseline clinical and demographic features of the cohorts are summarized in Table 1. In the patient samples, moderate symptoms (CTCAE grade 2) were more frequently observed in patients who had undergone ≥4 cycles of chemotherapy compared with < 4 cycles. Notably, severe symptoms (CTCAE grade 3) were only detected in patients that underwent ≥4 chemotherapy cycles. Therefore, the present study delineated the patient groups based on receiving < or > 4 cycles of chemotherapy to effectively capture the relationship between treatment duration and peripheral neuropathy severity. This grouping criterion reflects the increasing severity of CIPN with cumulative chemotherapy exposure (Ju et al., 2022). All patients provided written informed consent prior to sample enrollment, and the study protocol was approved by the Institutional Review Board of PNUH (approval number H-1803-014-065). The severity of CIPN was clinically evaluated by medical oncologists using version 4.03 of the National Cancer Institute Common Terminology Criteria for Adverse Events (U.S. Department of Health and Human Services, 2017).

**Table 1:** Baseline characteristics of patients with gastric cancer who underwent XELOX chemotherapy.

Variable	Group 1, *n* = 5	Group 2, *n* = 5
Sex, *n* (%)		
Male	2 (40)	4 (80)
Female	3 (60)	1 (20)
Age, years	60.4 ± 11.78	54 ± 10.1
Chemotherapy cycles, *n* (%)		
Cycle 2	1 (20)	
Cycle 3	4 (80)	
Cycle 4		1 (20)
Cycle 5		2 (40)
Cycle 6		2 (40)
NCI-CTCAE grade, *n* (%)		
Grade 1	4 (80)	
Grade 2	1 (20)	3 (60)
Grade 3		2 (40)
T, *n* (%)		
T1	1 (20)	1 (20)
T4a	4 (80)	3 (60)
T4b		1 (20)
N, *n* (%)		
N1		3 (60)
N2	2 (40)	1 (20)
N3a	1 (20)	
N3b	2 (40)	1 (20)
M, *n* (%)		
M0	5 (100)	4 (80)
M1		1 (20)

Values are presented as frequencies with percentages or means ± standard deviations. Group 1 includes patients receiving <4 cycles, and Group 2 includes those receiving 4-6 cycles. NCI-CTCAE; National Cancer Institute Common Terminology Criteria for Adverse Events; T, primary tumor; N, regional lymph node; M, distant metastasis; XELOX, capecitabine and oxaliplatin.

### 2.11 RT-qPCR analysis of clinical samples

Total RNA was extracted from patient blood samples using TRIzol Reagent (Invitrogen; Thermo Fisher Scientific, Inc.; cat. no. 15596026) according to the manufacturer’s protocol. cDNA was synthesized from total RNA using the High-Capacity cDNA Reverse Transcription Kit (Applied Biosystems; Thermo Fisher Scientific, Inc.; cat. no. 4368814) following the manufacturer’s instructions. mRNA expression levels of miR-378f, CAB39, Beclin-1 and LC3-II were quantified by RT-qPCR using the PowerUp SYBR Green Master Mix (Applied Biosystems; Thermo Fisher Scientific, Inc.; cat. no. A25742). Each 20 μL reaction consisted of 10 μL 2× PowerUp SYBR Green Master Mix, 2.5 μL each of forward and reverse primers and 5 μL cDNA template (10 ng). The thermal cycling conditions were as follows: UDG activation at 50˚C for 2 min, polymerase activation at 95˚C for 2 min, followed by 40 cycles of denaturation at 95˚C for 15 s, annealing at 55–60˚C for 15 s, and extension at 72˚C for 1 min. A dissociation curve analysis was performed at the end of each run to verify amplification specificity. The primer sequences used in the present study are listed in Table 2.

**Table 2:** Primer sequences used for RT-qPCR analysis.

Gene	Direction	Sequence (5’ → 3’)
LC3B	Forward	CGGTGAATAGAACGATACAAG
	Reverse	CTGAGATTGGTGTGGAGAC
Beclin-1	Forward	ACAGTGAACAGTTACAGATGGA
	Reverse	CTCAGCCTGGACCTTCTC
CAB39	Forward	CTGCGTCATCTTCCTCAGC
	Reverse	GCTGGGTTTCATGAAGGCAAA
β-actin	Forward	CTTCGCGGGCGACGAT
	Reverse	CCACATGGAATCCTCTGACC

CAB39, calcium-binding protein 39.

### 2.12 Statistical analysis

All experiments were conducted in triplicate, and the results are presented as the mean ± standard deviation. Statistical analyses were performed using GraphPad Prism 6 (Dotmatics), with data analyzed by one-way analysis of variance, followed by Tukey’s post-hoc test for multiple groups. *P* < 0.05 was considered to indicate a statistically significant difference.

## 3 Results

### 3.1 CAB39 as potential gene targeted by miR-378f

Bioinformatic analysis using miRNet was performed to identify common target genes among the five differentially expressed miRNAs associated with XELOX-induced peripheral neuropathy. The resulting interaction network comprised the five candidate miRNAs and their predicted target genes, with connecting lines representing miRNA-target interactions (Figure 1). Among the target genes identified, SEC24A and CAB39 were each targeted by at least three of the five candidate miRNAs. Specifically, SEC24A was targeted by hsa-mir-4666a-3p, hsa-mir-200c-3p and hsa-mir-27b-3p, whereas CAB39 was targeted by hsa-mir-200c-3p, hsa-mir-378f and hsa-mir-27b-3p. CAB39 was selected for further analysis because it was a predicted target of miR-378f, whose differential expression had been validated by RT-qPCR with statistical significance in our previous study (Ju et al., 2022).

**Figure 1:**
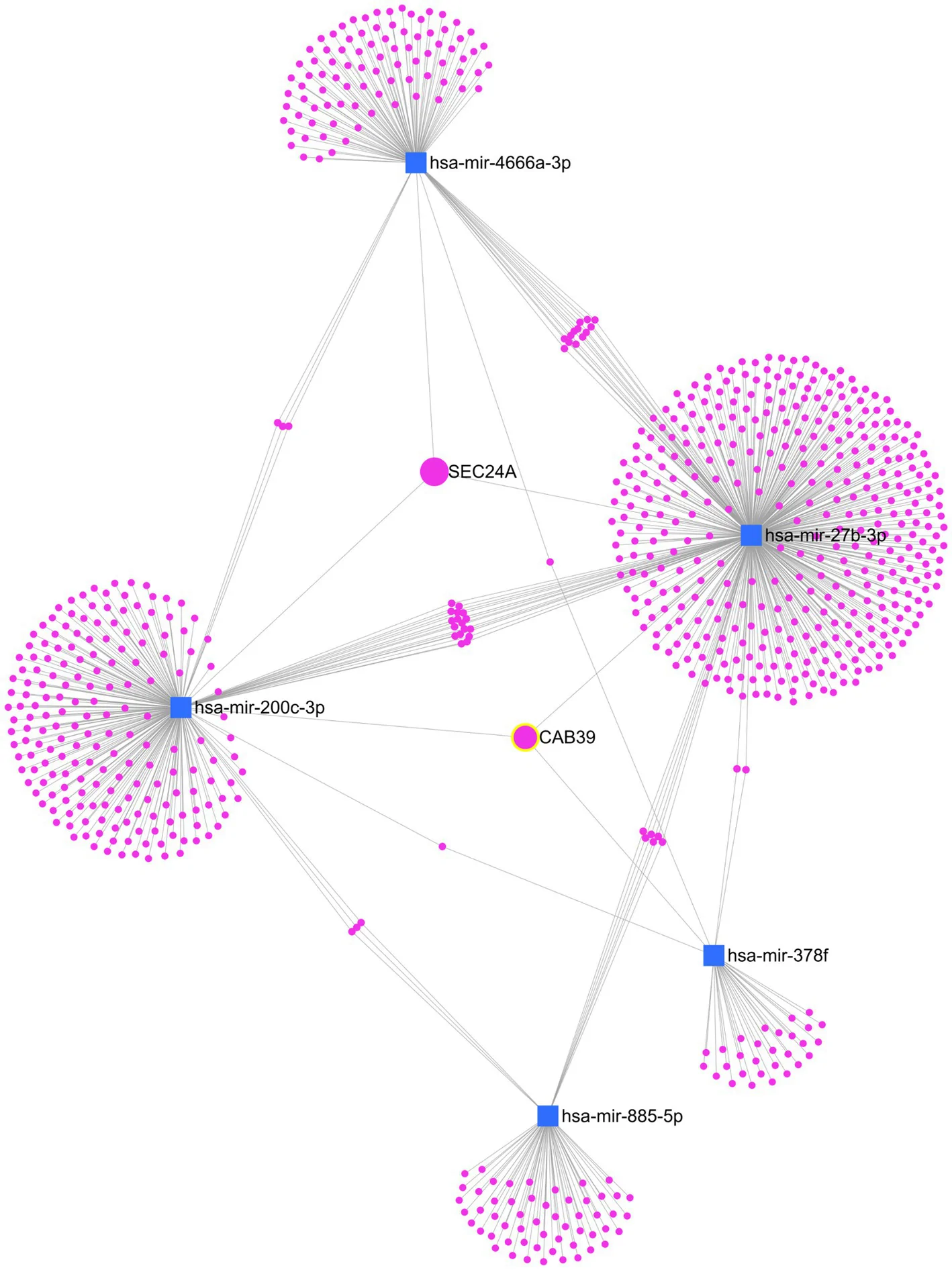
Predictive analysis of common target genes of differentially expressed miRNAs in patients treated with XELOX (capecitabine and oxaliplatin) chemotherapy. The network of differentially expressed miRNAs was predicted using miRNet. miRNA, microRNA.

### 3.2 miR-378f overexpression and suppression

To overexpress or knockdown miR-378f in the SH-SY5Y cell line, optimal transfection conditions were evaluated. A significant difference in miR-378f expression was found at 12 h post-Lipofectamine transfection with 30 μM of the miR-378f mimic (*P < *0.05). The highest miR-378f expression was observed at 24 h post-transfection (*P <* 0.001), followed by slightly decreased expression at 36 h post-transfection (*P <* 0.001; Figure 2a). The miR-378f mimic at 10 μM showed slightly overexpressed levels of miR-378f compared with the mimic-NC (*P <* 0.05). In addition, there was a clear difference in expression at the 30 μM dose (*P <* 0.001; Figure 2b). Therefore, transfections were performed under the 30 μM, 24 h condition. The cells were treated with the miR-378f inhibitor, which effectively suppressed miR-378f expression compared with the inhibitor-NC (*P <* 0.01), while the cells treated with the miR-378f mimic showed miR-378f overexpression compared with the mimic-NC (*P <* 0.001; Figure 2c).

**Figure 2:**
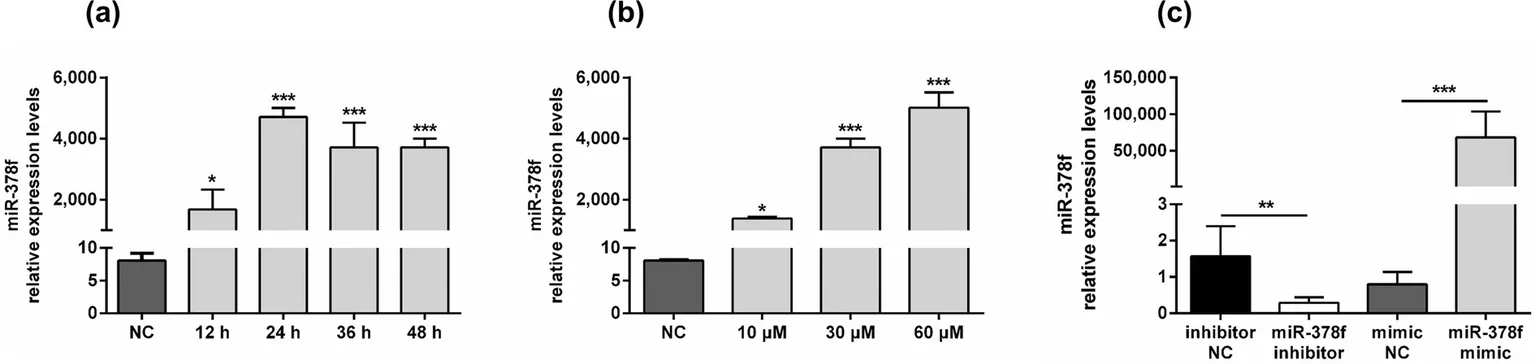
RT-qPCR analysis of relative post-transfection miR-378f levels. **(a)** Evaluation of transfection time using the miR-378f mimic. **(b)** Evaluation of transfection concentrations using the miR-378f mimic. **(c)** Relative expression of miR-378f after miR-378f mimic or miR-378f inhibitor transfection (*n* = 3/group; ^*^P < 0.05, ^**^P < 0.01, ^***^P < 0.001).

### 3.3 Neurite length affected by miR-378f expression

To investigate the effect of miR-378f expression on morphological changes in nerve cells, SH-SY5Y cells were observed under a microscope post-transfection (Figure 3a). Neurite length was extended by treatment with the miR-378f inhibitor (*P <* 0.001) and reduced by the miR-378f mimic (*P <* 0.001; Figure 3b). The number of branches extending from single cells was increased by the miR-378f inhibitor (*P <* 0.001) and slightly decreased by the miR-378f mimic, but the difference was not statistically significant (*P* = 0.2144; Figure 3c). In conclusion, the results show that miR-378f downregulation has a positive effect on neurite outgrowth in nerve cells.

**Figure 3:**
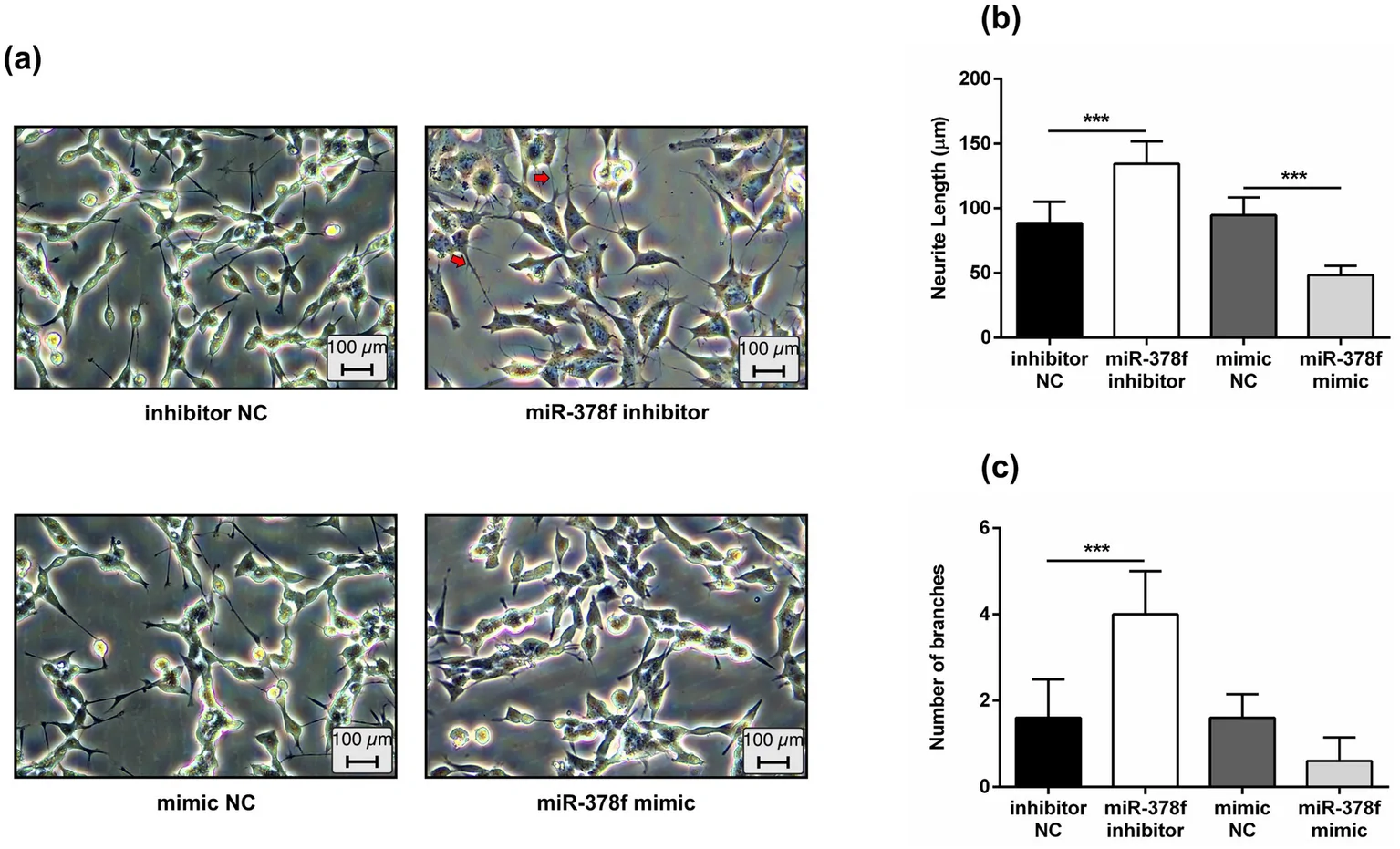
Morphological changes in SH-SY5Y human neuroblastoma cells according to miR-378f expression levels. **(a)** SH-SY5Y cellular morphology after miR-378f mimic and inhibitor transfection. Red arrows indicate elongated neurites (scale bar, 100 μm). **(b)** Measurement of neurite length. **(c)** Number of neurite branches (*n* = 5/group; ^***^P < 0.001).

### 3.4 Effect of miR-378f on autophagy regulation in nerve cells

To explore whether miR-378f regulates autophagy in nerve cells, SH-SY5Y human neuroblastoma cells were transfected with miR-378f mimics and inhibitors, and autophagy-related protein expressions were observed (Figure 4a). The expression of Beclin-1 protein was increased by miR-378f inhibition (*P <* 0.001) and decreased by miR-378f overexpression (*P <* 0.01; Figure 4b). By contrast, p62 protein expression was decreased by miR-378f inhibition (*P <* 0.05) and increased by miR-378f overexpression (*P <* 0.05; Figure 4c).

**Figure 4:**
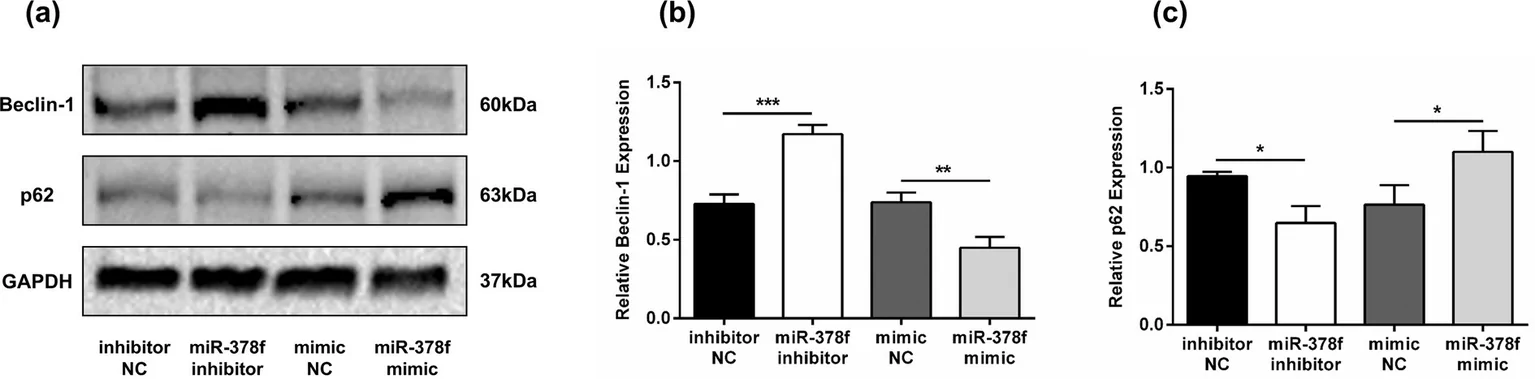
Changes in autophagy-related proteins in response to miR-378f expression levels. **(a)** Western blotting images of Beclin-1 and p62 in SH-SY5Y human neuroblastoma cells. **(b)** Relative expressions of Beclin-1 and **(c)** p62 after miR-378f transfection (*n* = 3/group; ^*^P < 0.05, ^**^P < 0.01, ^***^P < 0.001).

### 3.5 miR-378f directly targets CAB39

Based on the results shown in Figure 1, a STRING analysis was performed to investigate the interaction network associated with CAB39 and autophagy-related proteins (Figure 5). The analysis revealed associations between CAB39, AMPK, mTOR and several autophagy-related proteins, including Beclin-1, LC3, p62, unc-51-like autophagy-activating kinase 1 (ULK1) and ATG family proteins. Dual-luciferase reporter assays were performed to determine whether miR-378f directly targets CAB39 (Figure 6a). The co-transfection of firefly luciferase with CAB39 WT 3’-UTR/pmirGLO and the miR-378f inhibitor into SH-SY5Y cells increased luciferase activity compared with the negative control (*P <* 0.01). By contrast, co-transfection with the miR-378f mimic decreased luciferase activity compared with the negative control (*P <* 0.05; Figure 6b), whereas the co-transfection with CAB39 MUT 3’-UTR/pmirGLO showed no statistically significant difference between the miR-378f mimic and inhibitor (Figure 6c).

**Figure 5:**
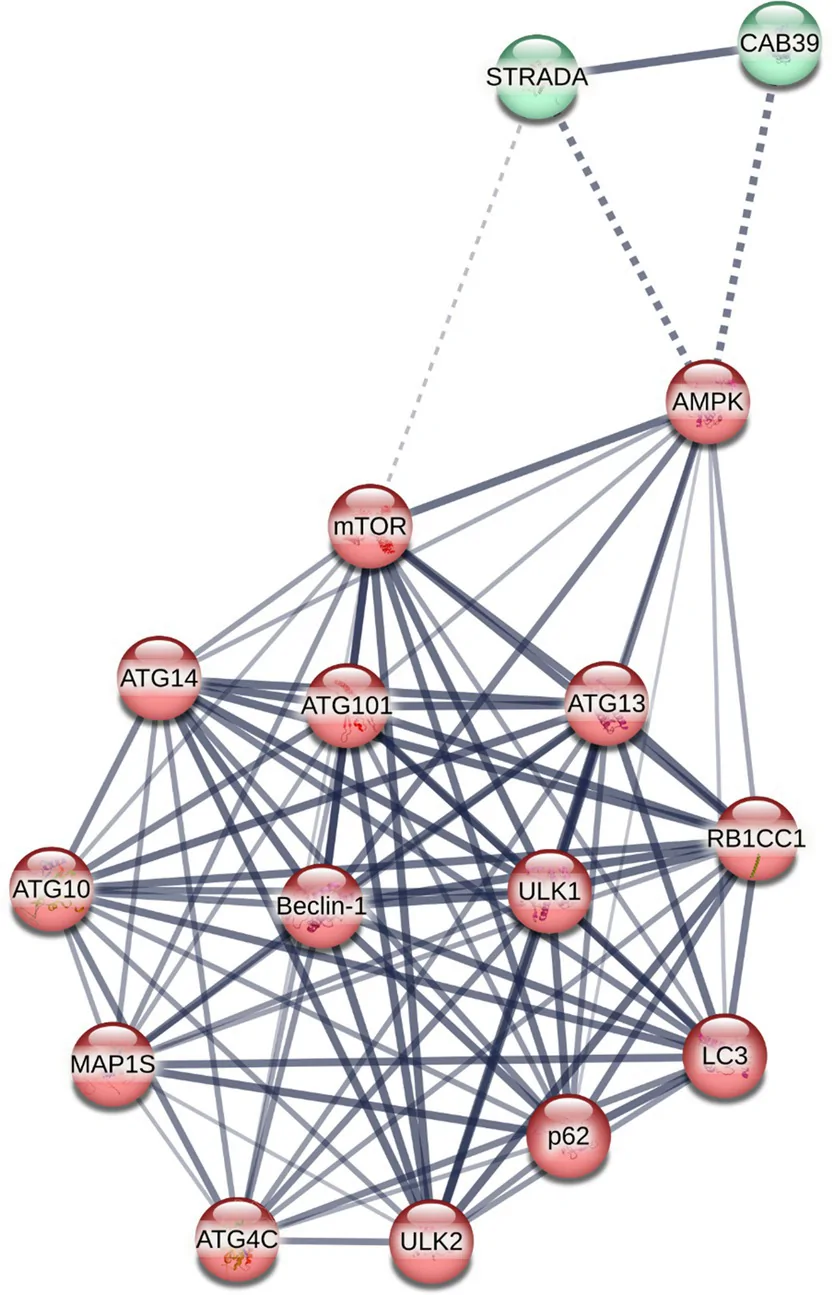
Prediction of target genes of miR-378f. Red indicates autophagy pathway, while green indicates energy-dependent regulation of mTOR by the LKB1-AMPK pathway.

**Figure 6:**
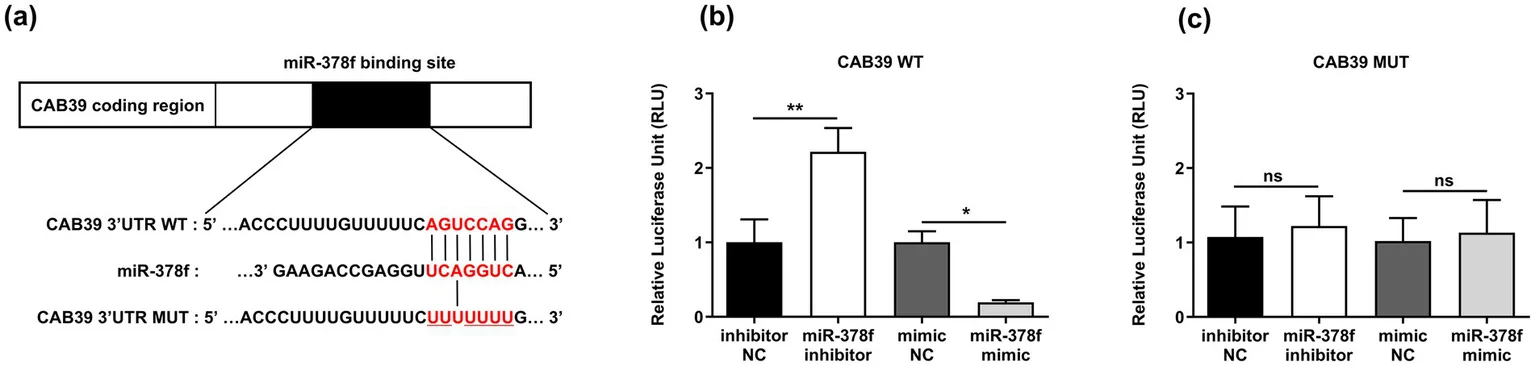
Prediction and validation of the target gene (CAB39) site for miR-378f. **(a)** miR-378f binding site in the 3’-UTR of CAB39. **(b)** Luciferase assay performed in SH-SY5Y cells co-transfected with CAB39 WT and miR-378f. **(c)** Luciferase assay performed in SH-SY5Y cells co-transfected with CAB39 MUT and miR-378f (*n* = 3/group, mutated sequences are underlined; ^*^P < 0.05, ^**^P < 0.01). ns, not significant; CAB39, calcium-binding protein 39; 3’-UTR, 3’-untranslated region; WT, wild type; MUT, mutant type.

### 3.6 miR-378f/CAB39 regulates AMPK/mTOR signaling and autophagy-related proteins

After demonstrating that miR-378f directly targets CAB39, western blotting was performed to examine the effects of miR-378f modulation on CAB39 expression, downstream AMPK/mTOR signaling, and autophagy-related proteins. Western blotting showed that treatment with the miR-378f inhibitor increased CAB39 expression levels (*P* < 0.05), while treatment with the miR-378f mimic suppressed CAB39 expression (*P* < 0.001; Figures 7a,b). Total AMPK expression levels showed no statistically significant differences between groups (inhibitor, *P* = 0.3920; mimic, *P* = 0.3262; Figure 7c). Similarly, total mTOR expression levels did not differ significantly between groups (inhibitor, *P* = 0.4837; mimic, *P* = 0.5244; Figure 7e). Phosphorylated AMPK expression was increased by treatment with the miR-378f inhibitor (*P* < 0.001) and decreased by treatment with the miR-378f mimic (*P* < 0.01; Figures 7a,d), while phosphorylated mTOR expression was decreased by treatment with the miR-378f inhibitor (*P* < 0.05) and increased by treatment with the miR-378f mimic (*P* < 0.05; Figures 7a,f). These findings suggest that miR-378f regulates AMPK/mTOR signaling through modulation of CAB39 expression.

**Figure 7:**
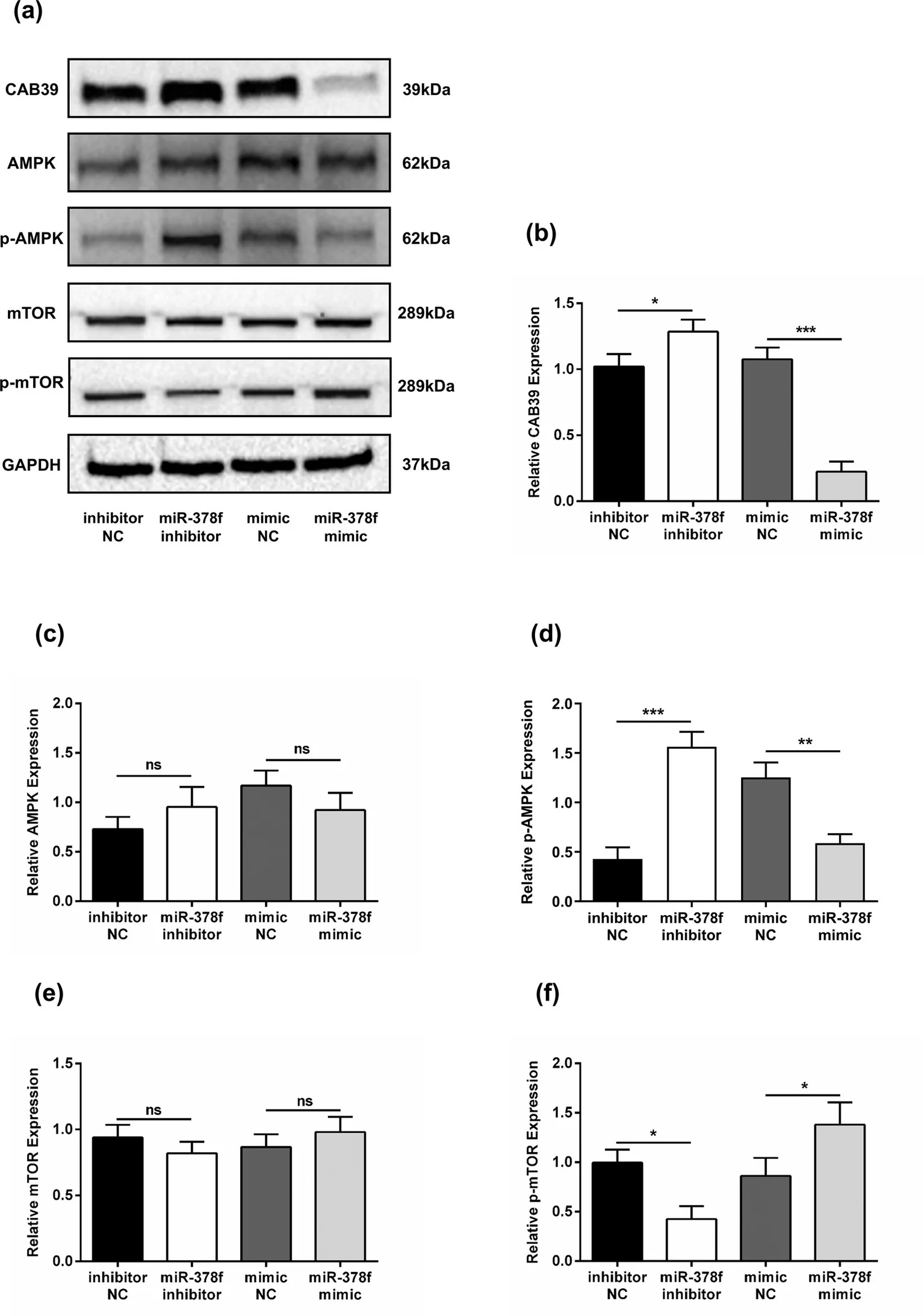
Identification of the CAB39-AMPK-mTOR pathway through western blotting. **(a)** Western blotting images of CAB39, AMPK, p-AMPK, mTOR and p-mTOR. **(b)** Relative expression of CAB39, **(c)** AMPK, **(d)** p-AMPK, **(e)** mTOR and **(f)** p-mTOR after co-transfection (*n* = 3/group; ^*^P < 0.05, ^**^P < 0.01, ^***^P < 0.001). ns, not significant; CAB39, calcium-binding protein 39; p, phosphorylated.

To further evaluate the downstream effects of miR-378f/CAB39 signaling, autophagy-related proteins were examined. Beclin-1 expression was increased by treatment with the miR-378f inhibitor (*P* < 0.001) and decreased by treatment with the miR-378f mimic (*P* < 0.01; Figures 8a,b), while p62 expression was markedly decreased after treatment with the miR-378f inhibitor (*P* < 0.001) and increased by treatment with the miR-378f mimic (*P* < 0.001; Figures 8a,c). The LC3-II/I ratio was increased after treatment with the miR-378f inhibitor (*P* < 0.001) and decreased by treatment with the miR-378f mimic (*P* < 0.01; Figures 8d,e). Together, these changes suggest that miR-378f downregulation contributes to the activation of autophagy-related molecular responses.

**Figure 8:**
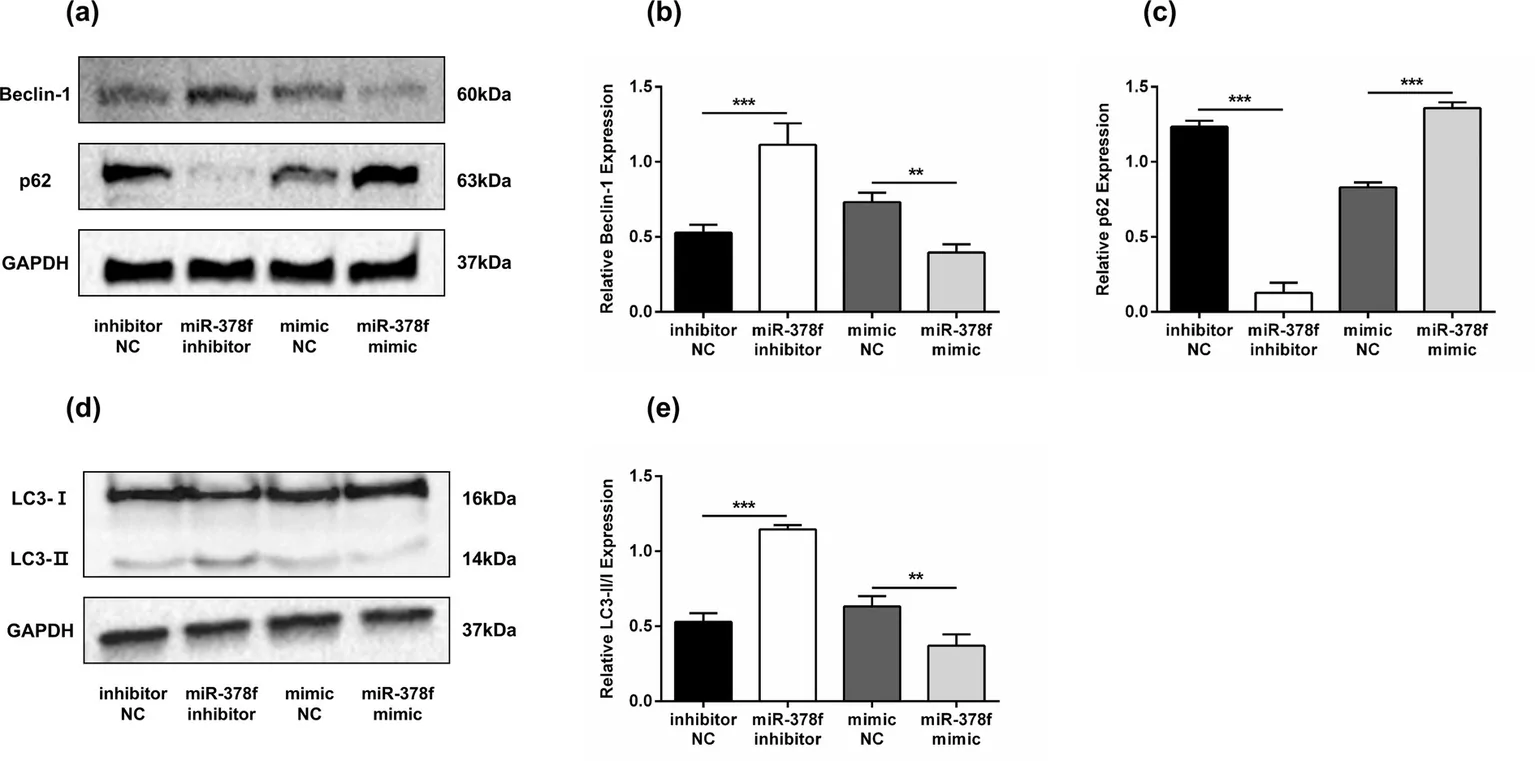
Identification of autophagy generation mechanisms by western blotting. **(a,d)** Western blotting images of Beclin-1, p62 and LC3-II/I after co-transfection. **(b,c,e)** Relative Beclin-1, p62 and LC3-II/I expressions. (*n* = 3/group; ^**^P < 0.01, ^***^P < 0.001). CAB39, calcium-binding protein 39.

### 3.7 Clinical validation of miR-378f regulation in gastric cancer patients with CIPN

RT-qPCR analysis of blood samples from patients with gastric cancer revealed distinct expression patterns based on XELOX chemotherapy cycles. miR-378f expression was significantly decreased in patients with higher-severity CIPN (Group 2, ≥4 cycles) compared with those with lower-severity CIPN (Group 1, <4 cycles). By contrast, CAB39 levels were markedly elevated in Group 2, alongside significant upregulation of autophagy markers Beclin-1 and LC3-II (Figure 9). These findings suggest that autophagy is enhanced as the severity of peripheral neuropathy increases, and is likely to facilitate peripheral nerve regeneration.

**Figure 9:**
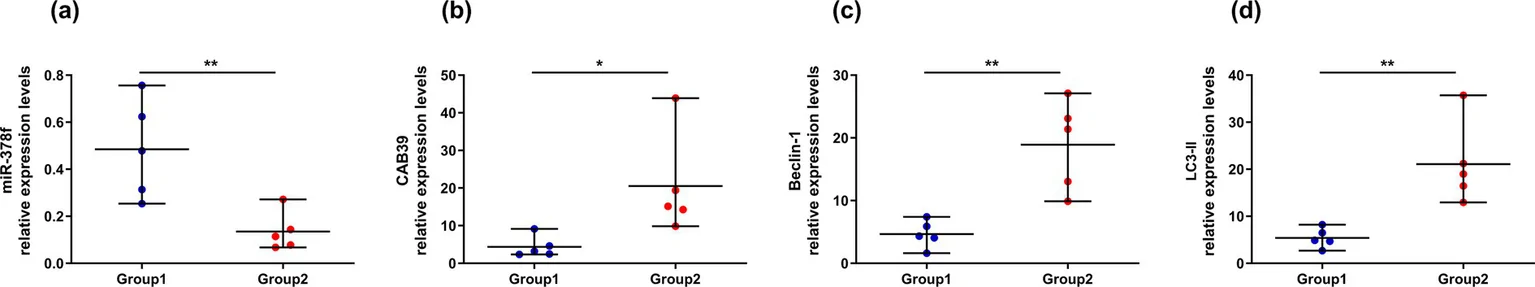
RT-qPCR analysis of miR-378f, CAB39 and autophagy markers in patients. **(a)** Relative miR-378f, **(b)** CAB39, **(c)** Beclin-1 and **(d)** LC3-II expression. Group 1 underwent <4 cycles of chemotherapy and Group 2 underwent ≥4 cycles of chemotherapy (*n* = 5/group; ^*^P < 0.05, ^**^P < 0.01). miRNA, microRNA; CAB39, calcium-binding protein 39.

## 4 Discussion

As advances in cancer treatment result in an increasing number of cancer survivors, long-term treatment-related complications that impair daily functioning and quality of life have become an important clinical concern (Thun et al., 2010; Mayer et al., 2017; Lustberg et al., 2023). CIPN develops frequently during chemotherapy and can persist for months or even longer after treatment, causing pain, numbness and tingling and potentially leading to dose reduction or premature discontinuation of chemotherapy (Mols et al., 2014; Colvin, 2019; Burgess et al., 2021). Meanwhile, therapeutic strategies based on miRNA mimics and inhibitors are being investigated in various diseases, highlighting the potential importance of identifying disease-associated miRNAs and their molecular targets (Ganju et al., 2017; Chakraborty et al., 2018; Condrat et al., 2020; Uzuner et al., 2022). Our previous study demonstrated that reduced miR-378f expression was associated with greater peripheral neuropathy severity in patients receiving XELOX chemotherapy (Ju et al., 2022). Therefore, the present study investigated the molecular targets of miR-378f and its potential relationship with autophagy-associated neuroregenerative responses in CIPN.

miR-451 reportedly inhibits glioma via PI3K/AKT pathway inhibition by targeting the CAB39 (Tian et al., 2012), whereas suppression of miR-107 protects osteoblasts from dexamethasone-induced damage by upregulating CAB39 expression and activating AMPK-Nrf2 signaling (Zhuang et al., 2020). These findings underscore the context-dependent roles of CAB39 and its regulation by various miRNAs in different cellular processes and pathologies. In the present study, CAB39 was identified as a potential target of miR-378f by bioinformatic analysis, and the direct interaction between miR-378f and the CAB39 3’-UTR was subsequently validated by dual-luciferase reporter assays. These findings support CAB39 as a molecular target through which miR-378f may regulate downstream signaling.

mTOR and AMPK are key regulators of autophagy and act as central sensors of cellular energy status and stress (Li and Chen, 2019; Wang et al., 2020). The dynamic interplay between AMPK and mTOR signaling determines whether autophagy-related pathways are activated or suppressed, as AMPK activation negatively regulates mTOR signaling while mTOR activation suppresses autophagy (Kim et al., 2011; Shang and Wang, 2011; Alers et al., 2012). As miRNAs generally suppress target gene expression at the post-transcriptional level, reduced miR-378f expression results in less repression of CAB39, thereby allowing CAB39 protein expression to increase. Consistent with this mechanism, miR-378f inhibition increased CAB39 expression, which was accompanied by increased AMPK phosphorylation and decreased mTOR phosphorylation, whereas miR-378f overexpression produced the opposite pattern. Together, these findings suggest that reduced miR-378f favors the activation of autophagy-related molecular responses through the CAB39/AMPK-mTOR pathway.

Autophagy is particularly relevant to neuronal repair because it contributes to the removal of damaged proteins and dysfunctional organelles that accumulate following neuronal injury (Maday, 2016). In peripheral nerves, activation of autophagy has also been associated with enhanced clearance of damaged cellular components and myelin debris, supporting axonal regeneration and functional recovery after injury (Huang et al., 2016; Yon et al., 2023). In the present study, miR-378f inhibition increased Beclin-1 expression and the LC3-II/I ratio while decreasing p62 expression. These molecular changes occurred in parallel with increased neurite outgrowth, supporting a potential association between autophagy-related responses and neuronal repair. However, direct autophagosome formation and autophagic flux were not measured in the present study; therefore, these findings should be interpreted as evidence of autophagy-associated molecular regulation rather than direct confirmation of increased autophagic flux.

In the context of CIPN, these findings suggest that activation of autophagy-associated signaling may represent an adaptive response to chemotherapy-induced neuronal stress. As illustrated in Figure 10, suppression of mTOR may facilitate downstream ULK1 activation and its interaction with Beclin-1, thereby promoting autophagosome formation, although ULK1 activation and autophagosome formation were not directly assessed in the present study (Park et al., 2018). Autophagy is a dynamic and context-dependent process; therefore, the present findings do not necessarily indicate that therapeutic induction of autophagy would be beneficial at all stages of CIPN. Instead, we propose that the reduction of miR-378f observed with greater CIPN severity may represent a compensatory molecular response that contributes to neuronal repair. Such compensatory responses are common in biological systems, where cellular stress can activate counter-regulatory pathways aimed at restoring homeostasis (Marder and Goaillard, 2006).

**Figure 10:**
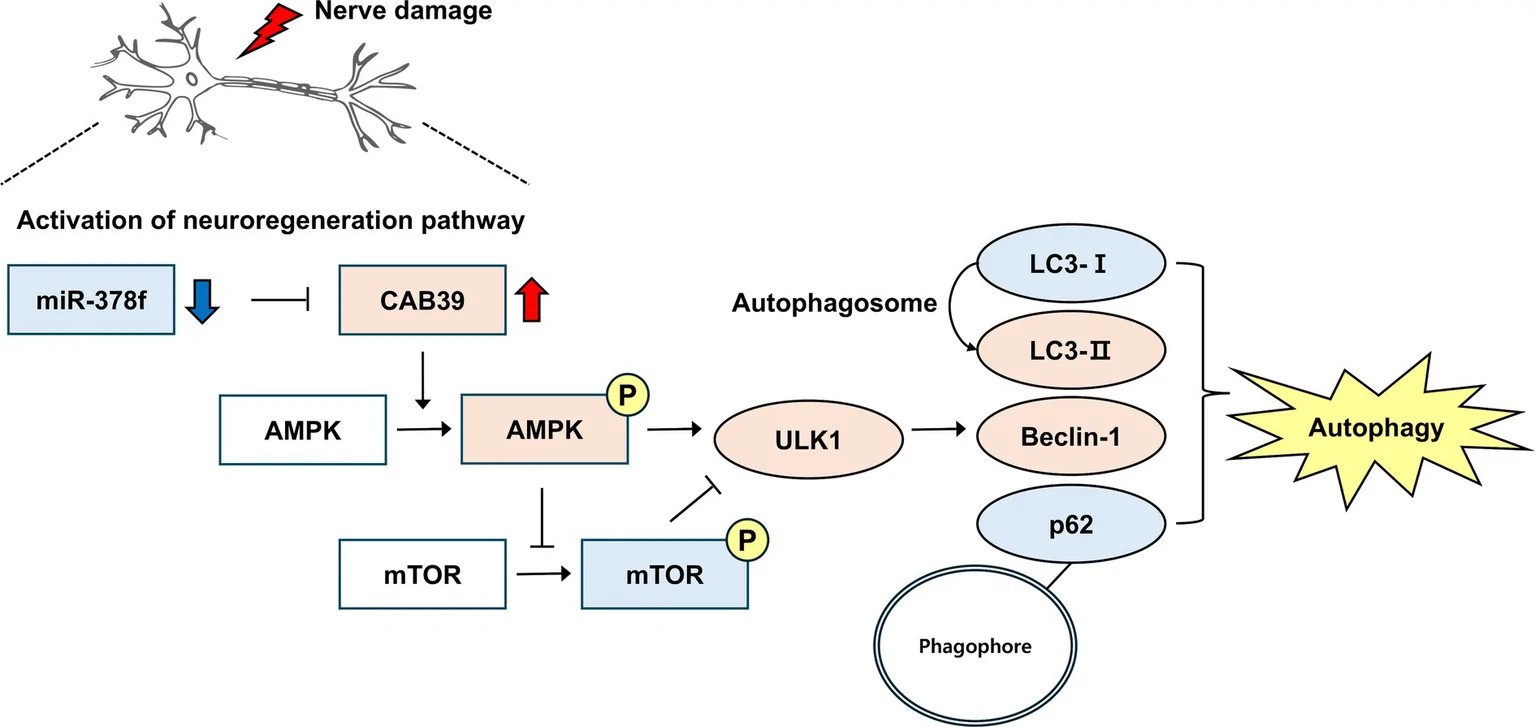
Schematic diagram illustrating the role of miR-378f in the regulation of autophagy in nerve cells. Blue indicates decreased expression, whereas red indicates increased expression. miRNA, microRNA.

The clinical findings further supported the relevance of this molecular pattern. Patients with greater chemotherapy exposure and a more severe distribution of CIPN showed lower miR-378f expression together with higher CAB39 and autophagy-related transcript levels. However, these findings should be interpreted cautiously because the clinical cohort was small and did not include a chemotherapy-exposed group without CIPN. In addition, SH-SY5Y cells are a neuroblastoma-derived neuronal model and do not fully reproduce the characteristics of primary peripheral sensory neurons affected in CIPN. The present study also did not directly expose neuronal cells to anticancer drugs, assess autophagic flux or autophagosome formation, or evaluate the proposed mechanism *in vivo*. Therefore, further studies using primary peripheral sensory neurons or other physiologically relevant neuronal models, direct assessment of autophagic dynamics, *in vivo* CIPN models, and larger independent patient cohorts will be required to establish the functional and therapeutic significance of this mechanism. Nevertheless, the present findings identify miR-378f/CAB39 regulation as a potential molecular link between AMPK/mTOR signaling, autophagy-associated responses, and neuronal repair in CIPN.

## Data Availability

The datasets presented in this study can be found in online repositories. The names of the repository/repositories and accession number(s) can be found in the article/Supplementary material.
