# Supplementary material for: miR-378f targets CAB39 to regulate autophagy in chemotherapy-induced peripheral neuropathy
Source: Front Mol Neurosci. 2026 Oct 7;19:1958322. doi: 10.3389/fnmol.2026.1958322 (PMC13683628; doi:10.3389/fnmol.2026.1958322)
Supplement: Supplementary file 1 [file Data_Sheet_1.PDF]

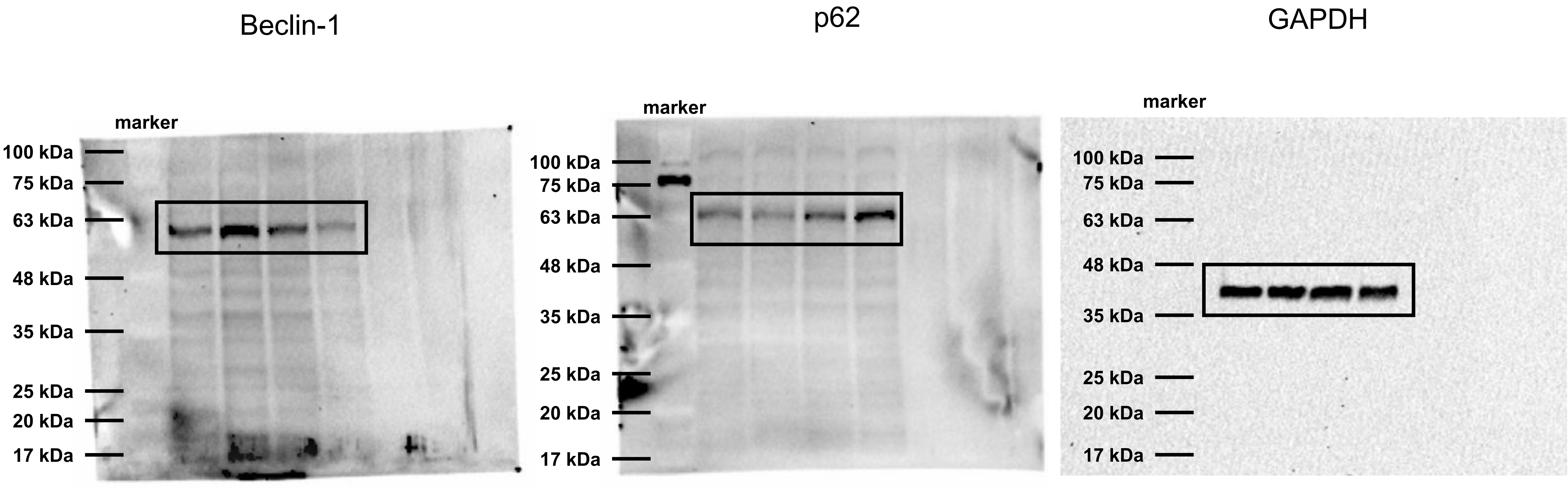


Original image of Western blot for Figure 4a Beclin-1, p62 and GAPDH protein expression in SH-SY5Y.

Ladder cat no. 24052 (iNtRON Bio, Seongnam, Republic of Korea)

CAB39 AMPK p-AMPK

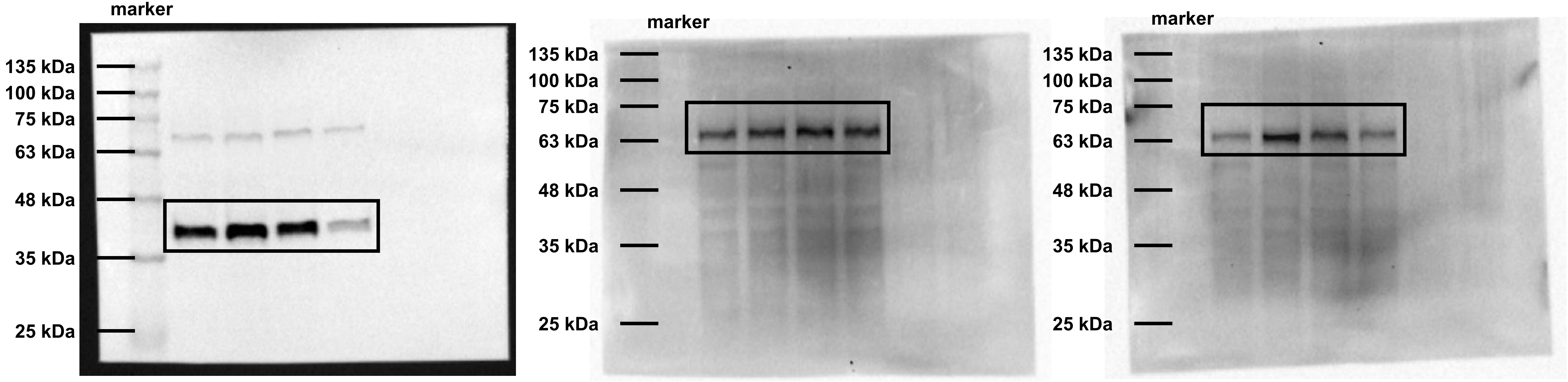


Original image of Western blot for Figure 7a CAB39, AMPK and p-AMPK protein expression in SH-SY5Y.

Ladder cat no. 24052 (iNtRON Bio, Seongnam, Republic of Korea)

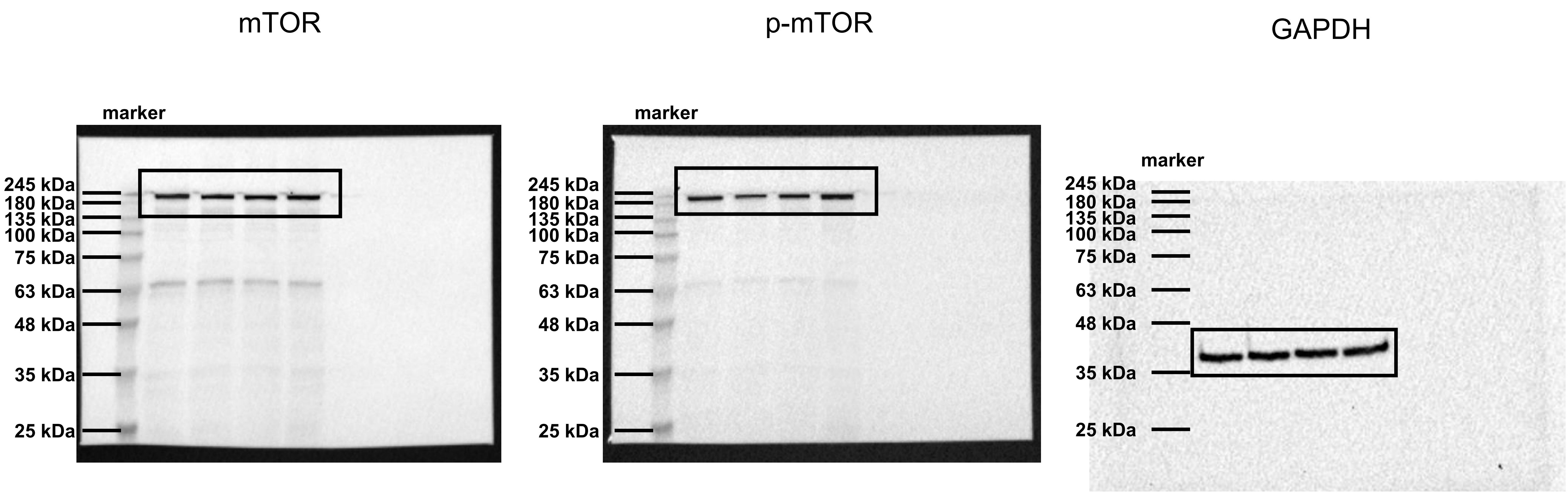


Original image of Western blot for Figure 7b mTOR, p-mTOR and GAPDH protein expression in SH-SY5Y.

Ladder cat no. 24052 (iNtRON Bio, Seongnam, Republic of Korea)

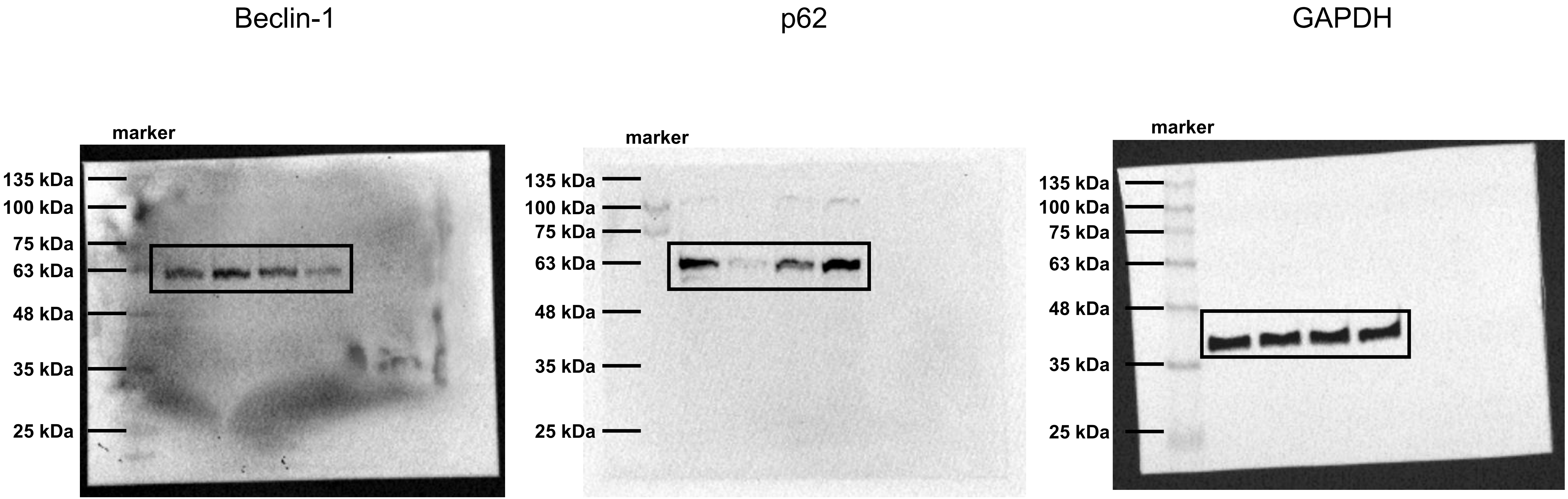


Original image of Western blot for Figure 8a Beclin-1, p62 and GAPDH protein expression in SH-SY5Y.

Ladder cat no. 24052 (iNtRON Bio, Seongnam, Republic of Korea)

LC3-II/I

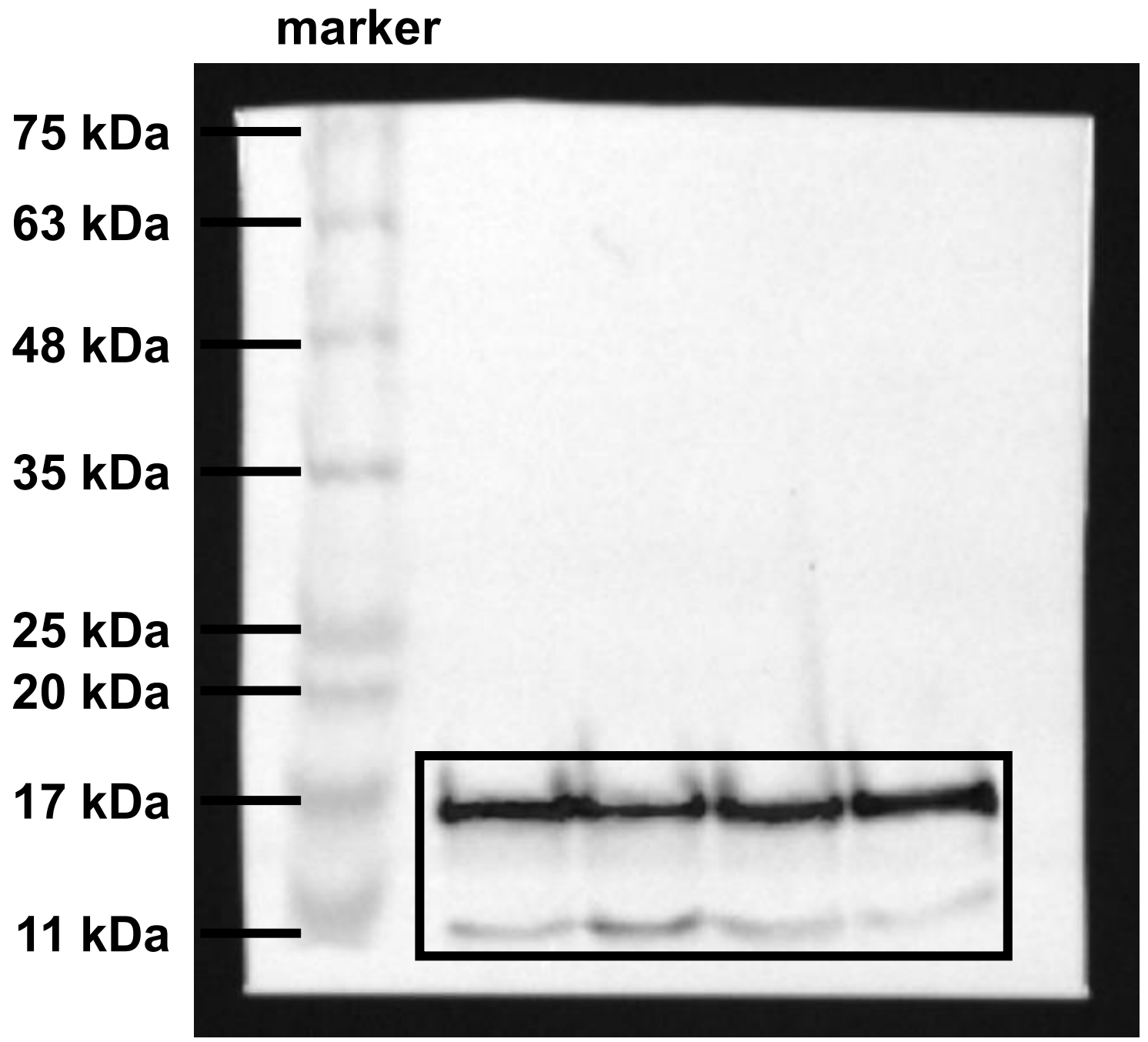


GAPDH

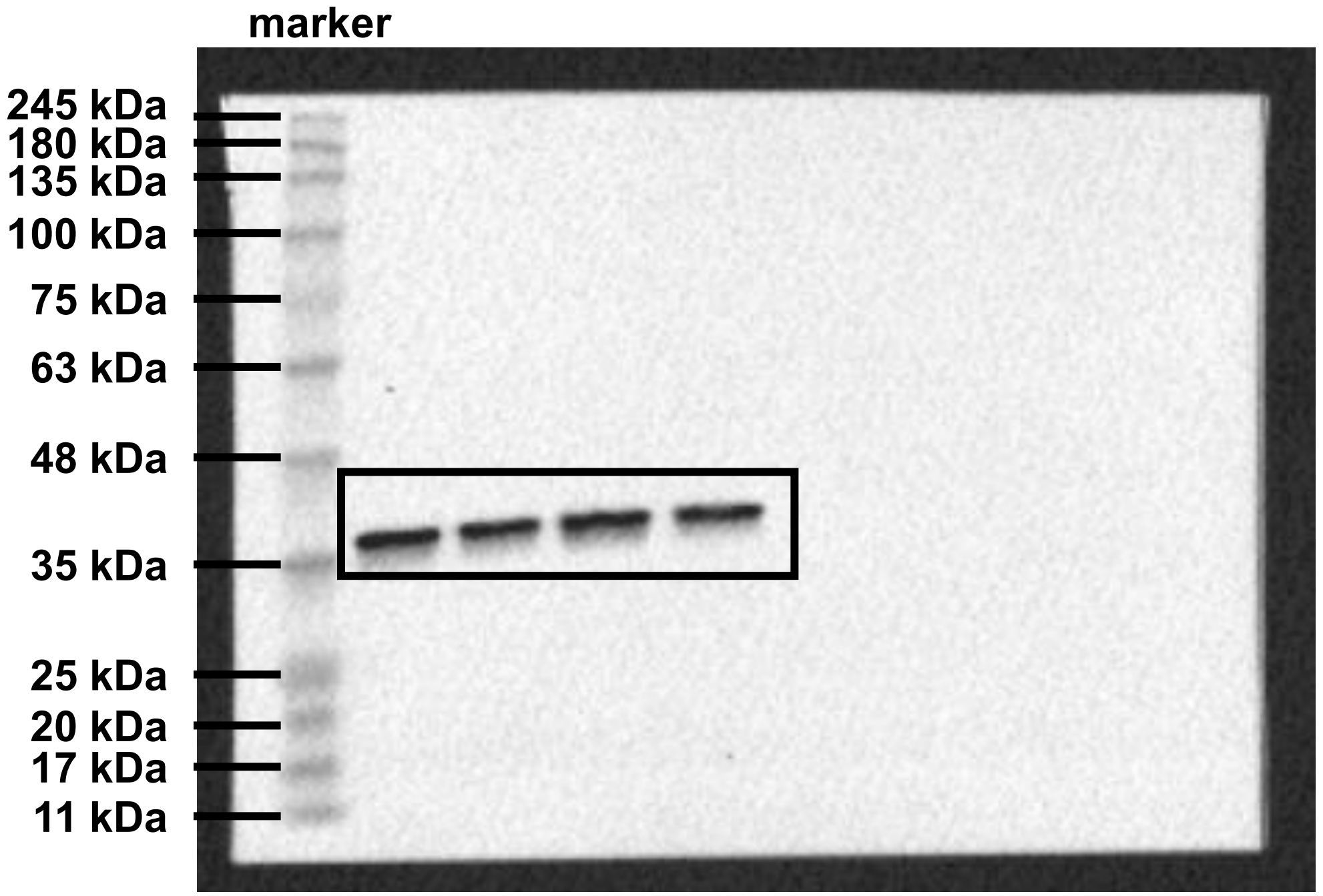


Original image of Western blot for Figure 8d LC3-II/I and GAPDH protein expression in SH-SY5Y.

Ladder cat no. 24052 (iNtRON Bio, Seongnam, Republic of Korea)
