# Supplementary material for: miR-378f targets CAB39 to regulate autophagy in chemotherapy-induced peripheral neuropathy
Source: Front Mol Neurosci. 2026 Oct 7;19:1958322. doi: 10.3389/fnmol.2026.1958322 (PMC13683628; doi:10.3389/fnmol.2026.1958322)
Supplement: Supplementary file 2 [file Table_1.DOCX]

Table S1. Representative miRNAs and their reported roles.

| **miRNA** | **Reported role/application** | **Reference** |
| --- | --- | --- |
| miR-221/222 | Circulating breast cancer biomarkers | Kim et al., 2019 |
| miR-124 | Neuronal differentiation; anti-inflammatory regulation | Das and Rao, 2022; Li et al., 2023 |
| miR-146a | Neuroinflammatory and innate immune regulation | Kim and Cho, 2021; Das and Rao, 2022; Li et al., 2023 |
| miR-155 | Pro-inflammatory response regulation | Das and Rao, 2022 |
| miR-21 | Schwann cell proliferation; axonal regeneration | Ning et al., 2020 |
| miR-1265 | CAB39-targeted suppression of oncogenic autophagy | Xu et al., 2019 |

CAB39, calcium-binding protein 39; miRNA, microRNA.
